# Advances in immune checkpoint inhibitor combination strategies for microsatellite stable colorectal cancer

**DOI:** 10.3389/fonc.2023.1112276

**Published:** 2023-02-02

**Authors:** Javier Ros, Francesca Balconi, Iosune Baraibar, Nadia Saoudi Gonzalez, Francesc Salva, Josep Tabernero, Elena Elez

**Affiliations:** ^1^ Medical Oncology, Vall d’Hebron Institute of Oncology (VHIO), Barcelona, Spain; ^2^ Oncologia Medica, Dipartimento di Medicina di Precisione, Università degli Studi della Campania Luigi Vanvitelli, Naples, Italy; ^3^ Medical Oncology, University Hospital and University of Cagliari, Cagliari, Italy

**Keywords:** colorectal cancer, immunotherapy, tyrosine kinase inhibitors, liver metastases, microsatellite stable (MSS)

## Abstract

Immune checkpoint inhibitors have reshaped the prognostic of several tumor types, including metastatic colorectal tumors with microsatellite instability (MSI). However, 90-95% of metastatic colorectal tumors are microsatellite stable (MSS) in which immunotherapy has failed to demonstrate meaningful clinical results. MSS colorectal tumors are considered immune-cold tumors. Several factors have been proposed to account for this lack of response to immune checkpoint blockade including low levels of tumor infiltrating lymphocytes, low tumor mutational burden, a high rate of WNT/β-catenin pathway mutations, and liver metastases which have been associated with immunosuppression. However, studies with novel combinations based on immune checkpoint inhibitors are showing promising activity in MSS colorectal cancer. Here, we review the underlying biological facts that preclude immunotherapy activity, and detail the different immune checkpoint inhibitor combinations evaluated, along with novel immune-based therapies, to overcome innate mechanisms of resistance in MSS colorectal cancer.

## 1 Introduction

Colorectal cancer (CRC) is the third most frequent cancer type diagnosed worldwide and the second cause of cancer-related deaths according to the Globocan data (1). Despite the improvement in early detection screening policies and the development of many novel treatments, in the USA, 5-year survival is only 12.5% (2), reflecting a clear unmet need for more effective treatments for patients with metastatic CRC (mCRC). Over the past decade, the development of immune checkpoint inhibitors has reshaped the prognostic of specific tumor types, notably melanoma and lung cancer, achieving deep, durable, and even complete responses. The identification of biomarkers of response is a much-needed paramount step forward in order to identify those patients who can benefit from immunotherapy strategies. In the case of mCRC, tumors with high microsatellite instability (MSI) or mismatch repair deficiency (MMRd), representing 5% of all mCRC, have achieved impressive and durable responses with immune checkpoint inhibitors. MSI tumors are highly enriched with neoantigens caused by the hypermutable status, typically presenting with high tumor mutational burden (TMB) and high levels of tumor infiltrating lymphocytes (TIL). Conversely, immune checkpoint inhibitors have demonstrated poor activity in most tumors that are mismatch-repair-proficient or microsatellite stable (MSS), which represent the vast majority (95%) of patients with mCRC.

MSI and hypermutated MSS tumors, also called “immune-hot tumors”, show high neoantigen loads and TMB, enriched activated CD4 and CD8 T cells, and conversely a low-frequency of myeloid-derived suppressor cells (MDSCs) and regulatory T cells (Tregs) and increased expression of CTLA-4 and PD-1 and of its ligand PD-L1. In the majority of MSS tumors, on the other hand, immunotherapy alone has demonstrated no clinical activity, and they have been characterized as being enriched for MDSCs, poorly infiltrated by T cells, and have downregulated checkpoint inhibitors, HLA class-I and class-II, and CD4 and CD8 T cells (3). MSS tumors are considered “immune-cold tumors” characterized by low TMB and a lack of immune cell infiltration, which have been positioned as the main mechanisms of immune resistance (4, 5). The neoantigen enrichment of MSI mCRC is related to the hypermutable status in the context of MSI whereas MSS tumors harbor features of chromosomal instability, in which genomic aberrations of the genomic structure occur on a larger scale, leading to lower TMB and neoantigen generation (6). There is evidence suggesting that survival benefit among patients with CRC and high-TMB is limited to MSI and POL-E or POL-D1 tumors meaning that very few patients with MSS/high-TMB benefit from immune checkpoint blockade (7).

Overall, the lack of response to immune checkpoint inhibitors among patients with MSS mCRC may be driven by several molecular and histopathological factors. Mutations in the WNT/β-catenin signaling pathway genes, which is frequent in CRC, as well as liver metastases, may induce antitumor immunity inhibition and immune tolerance leading to T cell exclusion, explaining the upfront lack of response to immunotherapy. Furthermore, several clinical trials have demonstrated that patients with MSS mCRC without liver metastases are more prone to respond to immune checkpoint inhibitor combinations (8, 9). Several of the strategies attempting to increase immune checkpoint inhibitor activity in the MSS mCRC population have unfortunately given poor results, however, novel combinations appear promising. In this review, we describe the underlying biological features leading to MSS mCRC innate resistance to immune checkpoint inhibitors as well as the current clinical approaches and future directions to improve outcomes with immune checkpoint inhibitors in MSS mCRC patients.

## 2 The biological background limiting immune checkpoint inhibitor activity in MSS mCRC

### 2.1 Tumor cell infiltrate regulates immune response

**Figure 1:**
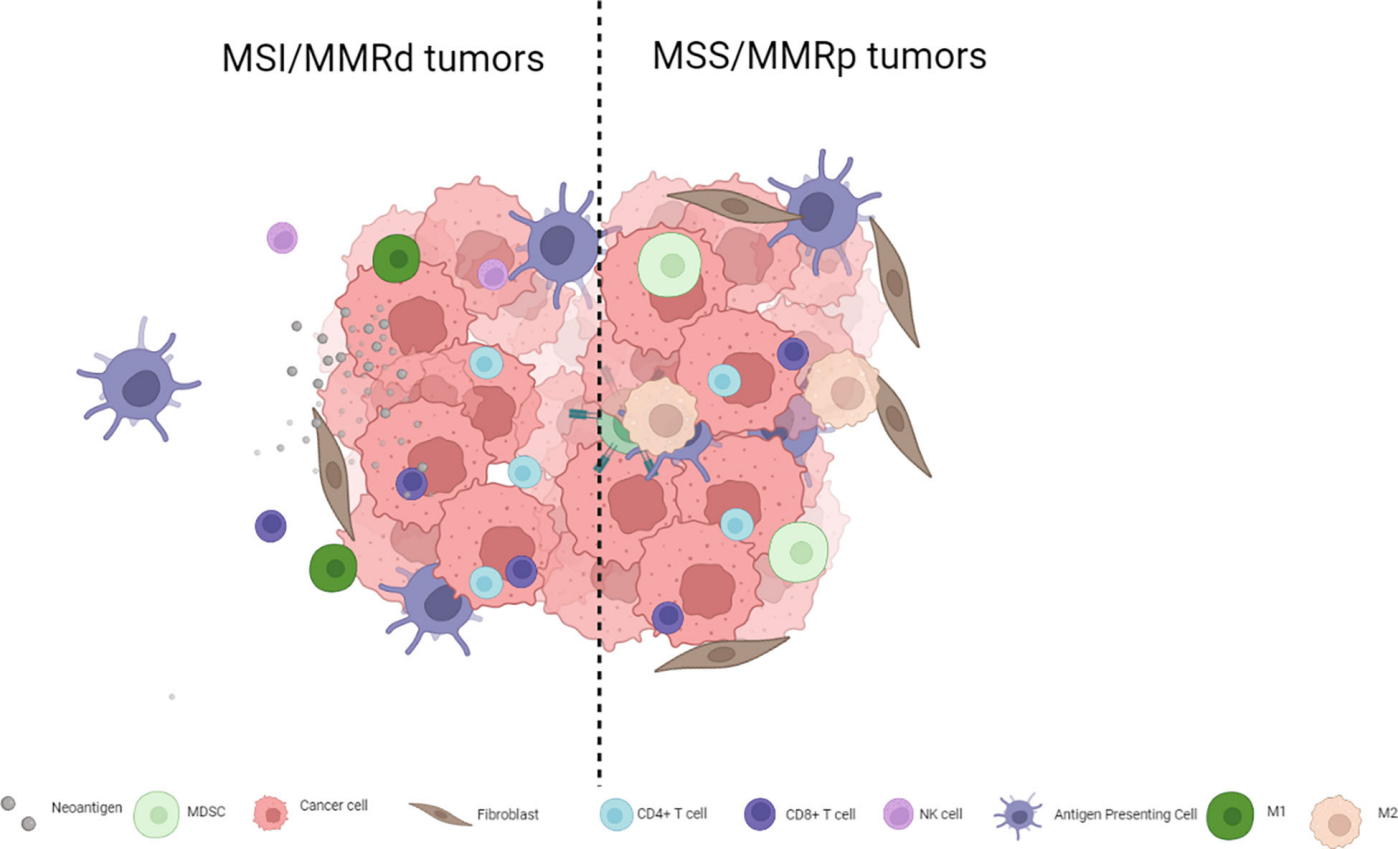
Key differences in immune cell infiltration for MSS and MSI mCRC.

The immunotherapy strategies tested to date have shown limited antitumor activity in MSS mCRC for multiple reasons including, among others, low TMB and low immune infiltration compared to MSI colorectal tumors or other hypermutated subtypes such as POL-E or POL-D mutated tumors. MSI tumors are inflamed and infiltrated with lymphocytes, T helper 1 cells, CD4+ cells, and macrophages and have a microenvironment rich in type I interferons (4, 10, 11), whereas their MSS counterparts are not inflamed and are usually PD-L1-negative (5, 12). Not only do MSS colorectal tumors have fewer TILs and lower TMB but their genomic alterations also seem to be poorly neo-antigenic (13). Thus, switching a tumor from an immune cold phenotype that abrogates T cell activity to a hot phenotype that leads to lymphocyte activation and tumor infiltration may improve immune checkpoint inhibitor efficacy. The complexity of MSS mCRC goes far beyond simple biomarkers such as PD-L1 expression, and TIL or TMB levels. **Figure 1** presents the main differences between MSS and MSI mCRC.

Cell infiltration plays a paramount role regulating immune checkpoint inhibitor activity. MDSCs are immature myeloid cells found in several tumor types. In CRC, tumor cells promote the induction of MDSCs that have an immunosuppressive role which facilitates tumor growth and spread by releasing factors such as reactive oxygen species, TGFβ, and nitric oxide (14). MSI and hypermutated tumors show a depletion of MDSCs and Tregs, unlike in MSS tumors (3). Furthermore, patients with a high level of MDSCs have shorter progression-free survival (PFS) with chemotherapy (15). MDSCs have been demonstrated to inhibit T cell proliferation, while blocking MDSC function was shown to restore INFγ secretion by T cells (16). In addition to MDSCs, tumor-associated macrophages (TAMs) also play a fundamental role in immune system modulation. TAMs can be divided into two major groups based in their phenotype. M1 macrophages are involved in tumor growth by secreting pro-inflammatory cytokines such as TNF-alfa, IL-1-B or IL-12, driving a Th1 response. On the other hand, M2 macrophages secrete immunosuppressive cytokines such as IL-10 and TGFβ which promote tumor progression and metastases. However, unlike other tumor types, the prognostic impact of TAMs in CRC remains unclear, likely in part because of high plasticity between macrophage subsets and a lack of well-defined markers to properly identify them (17–20). Likewise, the exact role of Tregs in CRC has not been fully elucidated. Overall, Tregs have a pro-inflammatory role, secreting TGFβ and IL-10, and suppressing CD4 and CD8 T cells and downregulating IFNγ and IL-2 secretion in tumors leading to an immunosuppressive microenvironment (21, 22).

### 2.2 CRC genomic drivers result in an immunosuppressive microenvironment

Whereas MSI CRC tumors are enriched for hypermethylation, and *BRAF* and *RNF43* mutations, MSS tumors are enriched in *APC* and *KRAS* mutations and chromosomal instability. These molecular features reflect the differences in the carcinogenesis pathway that may contribute to responses to immune checkpoint blockade strategies (23). β-catenin oncoprotein is a paramount activator of WNT signaling in CRC, particularly *APC* mutations (present in more than 70% of MSS CRC but in only 20% of MSI CRC) that regulate the function of β-catenin (<ξ>)(24). Studies in melanoma identified that increased β-catenin activation resulted in a decreased CD8 and CD103 dendritic cell population that hampered T cell recruitment into the tumor microenvironment, leading to a T cell exclusion status. Activated WNT/β-catenin signaling has been inversely correlated with the absence of T cell infiltration (CD8+ cells, CD45RO+ cells, and CD3+ cells but not with FOXP3+ cells) regardless of mutational load, and nuclear CTNNB1 protein expression (a marker of activation of the WNT pathway) was inversely associated with TIL levels in CRC samples. Non-inflamed CRC tumors were enriched in β-catenin signaling pathway (23, 25, 26), thus suggesting that WNT signaling inhibitors may be able to reverse immune exclusion (20). Furthermore, MSS tumors that harbor a lower mutational load exhibit less cancer immunoediting along with higher WNT pathway signaling (including *RNF43* and *Axin* 1/2 mutations, and R-spondin gene fusions) and decreased T cell infiltration leading to “cold tumors” that are less responsive to immunotherapy strategies. Furthermore, *APC* biallelic mutations are associated with increased WNT signaling and decreased TILs in both MSS and MSI CRC. This finding is of particular interest considering that up to 70% of all CRC cases have *APC* biallelic loss. Finally, hypomethylation of AXIN2 super-enhancers has been associated with decreased TILs regardless of *APC* alterations in MSS tumors. Taken together, these data suggest there are multiple pathways that limit immune response in cancers with active WNT signaling (23, 27, 28).

Transforming growth factor-β (TGF-β) signaling is a well-known oncogenic pathway driving CRC; it acts as an oncogenic factor by creating an immune-suppressive tumor microenvironment, and inducing tumor cell proliferation, angiogenesis, and metastatic spread (29). TGF-β levels strongly correlate with consensus molecular subtype (CMS) 4, the mesenchymal subtype characterized by stromal infiltration and epithelial-mesenchymal transition based on genomic profiles. Increased TGF-β is associated with increased regulatory T cells and downregulation of natural killer cells, leading to an immunosuppressive microenvironment (29, 30). Whereas 76% of samples in CMS1 tumors -the inflamed subgroup- were MSI, among CMS4 tumors, 92% were MSS and 8% were MSI. Furthermore, TGF-β has been noted in liver metastases in which CD4 and CD8 T cells were downregulated and immune tolerance is abrogated by liver type 1 macrophage activation and a high number of Treg (31–33). This can partially explain what has been observed in some clinical trials in which patients with liver metastases show lower response rates with immune checkpoint inhibitors compared with patients without liver metastases (8, 9, 34–36). In addition, preclinical inhibition of TGF-β resulted in reduced immune-evasion process (37).

Finally, alterations in the mitogen-activated protein kinase pathway (MAPK) are common events in CRC, particularly *RAS* mutations (50%) and *BRAF* mutations (10%). Mutations in both *RAS* and *BRAF* perpetuates kinase activation, leading not only to cell growth, invasion, and metastases, but also to tumor microenvironment differentiation, and have been shown to reduce T cell CD8+ infiltration and diminish neoantigen presentation by impairing the interferon pathway. Nevertheless, treatment with either BRAF or KRAS G12C inhibitors led to reduced MDSC infiltration and increased TILs (38–41). Current studies combining a BRAF inhibitor with an anti-PD-1 have shown deep clinical impact, with prolonged PFS among patients with a *BRAF*-V600E mutation (42–44). Finally, while the predictive value of PD-L1 has been demonstrated in several tumor types, this has not been the case in CRC in either the CHECKMATE-142 trial with nivolumab plus ipilimumab, or the phase I trial with pembrolizumab (5, 12). The efficacy of combined pembrolizumab plus co-formulated favezelimab, in patients with PD-L1-positive refractory CRC is currently being evaluated in a phase III trial (NCT05064059).

## 3 Strategies to overcome mechanisms of resistance to immune checkpoint inhibitor strategies in MSS mCRC

The following section reviews the large body of research that has focused on strategies to identify and overcome the mechanisms of resistance to immune checkpoint inhibitors in MSS CRC. A summary of completed and ongoing clinical evaluations according to treatment strategy in MSS CRC is presented in **Tables 1**, **2** respectively, covering a range of settings with frontline, refractory, and maintenance therapies.

**Table 1:** Completed clinical trials evaluating therapeutic strategies with immune checkpoint inhibitors in MSS mCRC.

STRATEGY	STUDY	PHASE	SETTING	TREATMENT	SAMPLE SIZE	ORR (%)	PFS (MONTHS)	OS (MONTHS)	REFERENCE
Immunotherapy	Keynote-016 NCT01876511	II	Refractory	Pembrolizumab	18	0	2.2	5	(45)
	CheckMate-142 NCT02060188	II	First-line	Nivolumab-Ipilimumab	23	NA	1.4	NA	(46)
	CCTC-CO.26 NCT02870920	II	Refractory	Durvalumab-Tremelimumab/BSC	119/61	0.5/0	1.8/1.9	6.6/4.1	(47)
	NCT02720068	I	Refractory	Pembrolizumab-Favelizumab	80	6.3	2.1	NA	(48)
	C-800 NCT03860272	FIH	Refractory	Botensilimab-Balstilimab	41	24	NA	NA	(8)
	NCT03156114	I	Refractory	BI754111-BI754091	40	7.5	NA	NA	(49)
	Imblaze-370 NCT02788279	III	Refractory	Atezolizumab	90	2	1.9	7.1	(50)
MEK inhibitors + ICI	Imblaze-370 NCT02788279	III	Refractory	Atezolizumab-Cobimetinib	183	3	1.9	8.9	(50)
Tyrosine kinase inhibitors + ICI	REGONIVO NCT03406871	Ib	Refractory	Regorafenib-Nivolumab	24	33	7.9	NR	(51)
	NCT03712943	Ib	Refractory	Regorafenib-Nivolumab	52	8	4.3	11.1	(34)
	NCT04126733	II	Refractory	Regorafenib-Nivolumab	70	7	1.8	12	(52)
	NCT03657641	I/II	Refractory	Regorafenib-Pembrolizumab	73	0	2	10.9	(53)
	REGOMUNE NCT03475953	II	Refractory	Regorafenib-Avelumab	48	0	3.6	10.8	(54)
	NCT04362839	I	Refractory	Regorafenib-Ipilimumab-Nivolumab	29	31	4	19.6	(9)
	CAMILLA NCT03539822	II	Refractory	Cabozantinib-Durvalumab	36	28	4.4	9.1	(55)
	COSMIC-021 NCT03170960	Ib	Refractory	Cabozantinib-Atezolizumab	31	9.7	3	14	(56)
	NCT03332498	I/II	Refractory	Ibrutinib-Pembrolizumab	40	0	1.4	6.6	(57)
	LEAP-005 NCT03797326	II	Refractory	Lenvatinib-Pembrolizumab	32	22	2.3	7.5	(58)
EGFR inhibitors + ICI	SWOG2107 NCT04017650	I/II	Refractory	Nivolumab-Encorafenib-cetuximab	26	45	7.3	11.4	(42)
	LCCC1632 NCT03442569	II	Refractory	Nivolumab-Ipilimumab-Panitumumab	56	5	5.7	NA	(59)
	CAVE NCT04561336	II	Refractory	Avelumab-Cetuximab	71	8.5	3.6	11.6	(60)
	AVETUX NCT03174405	II	First-line	FOLFOX-Cetuximab-Avelumab	43	79.5	11.5	NA	(61)
Chemotherapy + ICI	AtezoTRIBE NCT03721653	III	First-line	Atezolizumab-FOLFOXIRI-Bevacizumab/FOLFOXIRI-Bevacizumab	132/67	59/64	12.9/11.4	NA	(62)
	BACCI NCT02873195	II	Refractory	Atezolizumab-Capecitabine-Bevacizumab/Capecitabine-Bevacizumab	82	10/5	5/3.3	10.3/10.2	(63)
	CHECKMATE-9X8 NCT03414983	II	First-line	FOLFOX-Bevacizumab-Nivolumab vs FOLFOX-Bevacizumab	157/68	60/46	11.9/11.9	29.2/NR	(64)
	MAYA NCT03832621	II	Refractory	Temozolomide+Nivolumab+ipilimumab	33	45	7	18.4	(65)
	MEDITREME NCT03202758	II	First-line	FOLFOX-Durvalumab-Tremelimumab	57	61	8.4	NA	(66)
	MODUL NCT02291289	II	First-line	FOLFOX-Bevacizumab-Atezolizumab/FOLFOX-Bevacizumab	297/148	NA	7.2/7.4	22/22	(67)
Bispecific antibodies	NCT02324257	I	Refractory	Cibisatamab	68	6	NA	NA	(68)
	NCT02650713	I	Refractory	Cibisatamab-Atezolizumab	38	12	NA	NA	(68)
Radiotherapy	NCT03122509	II	Refractory	Radiotherapy+Durvalumab+Tremelimumab	24	8.3	1.8	11.4	(69)
	NCT03104439	II	Refractory	Radiotherapy+Nivolumab+Ipilimumab	40	12.5	NA	NA	(70)
Vaccines	NCT01413295	II	Refractory	Dendritic cell vaccine/BSC	28/24	0/0	2.7/2.3	6.2/4.7	(71)
	FXV	II	First-line	HLA-A*2402-restricted peptides+ oxaliplatin-based chemotherapy	50 HLA-A*2402-matched/46 HLA-A*2402-unmatched	62/60.9	7.2/8.7	20.7/24	(72)
Intratumoral	NCT00149396	I/II	Refractory	NV1020 hepatic artery infusion+chemotherapy	22	4.5	6.4	11.8	(73)

BSC, best supportive care; FIH, first in human; ICI, immune checkpoint inhibitor; NA, not available; NR not reached; ORR overall response rate; OS, overall survival; PFS, progression-free survival.

**Table 2:** Novel therapeutic strategies in ongoing clinical studies in MSS mCRC.

Strategy	Study ID	Phase	Setting	Treatment combination
Immunotherapy plus tyrosine kinase inhibitors	NCT03539822	II	refractory	Cabozantinib-durvalumab
	NCT04776148	III	refractory	Pembrolizumab-lenvatinib
	NCT04776148	II	refractory	Lenvatinib-durvalumab
Immunotherapy plus chemotherapy	NCT04068610	Ib/II	first line	FOLFOX-bevacizumab+/-durvalumab-oleclumab
	NCT04262687	II	first line	Pembrolizumab-CAPOX-bevacizumab
	AVETRIC NCT04513951	II	first line	FOLFOXIRI-avelumab-cetuximab
	ARETHUSA NCT03519412	II	refractory	Temozolomide then pembrolizumab
	NIVACOR NCT04072198	II	first-line	FOLFOXIRI-bevacizumab-nivolumab
Immunotherapy plus anti-EGFR	NCT03442569	II	first line	Nivolumab-ipilimumab-panitumumab
Immunotherapy plus PI3CA inhibitor	NCT03177058	I/II	refractory	Nivolumab-copanlisib
Immunotherapy plus anti-LAG3	NCT03642067	II	refractory	Relatlimab-nivolumab
Immunotherapy plus WNT/β-catenin inhibitor	NCT02675946	I	refractory	CGX1321-pembrolizumab
	NCT04907539	II	refractory	RXC004 +/- Nivolumab
Immunotherapy plus TGF-β inhibitor	NCT04952753	II	second-line	NIS793-chemotherapy
Immunotherapy combinations	NCT03860272	I	refractory	Botensilimab-balstilimab
Immunotherapy plus radiotherapy	NCT029929112	II	refractory	Atezolizumab-radiotherapy
	NCT02437071	II	refractory	Pembrolizumab-radiotherapy
Immunotherapy plus MAPK inhibitor	CodeBreaK 100 NCT03600883	I/II	refractory	AMG510+/-Anti-PD1/anti-PD-L1
	NCT04017650	II	refractory	Encorafenib-cetuximab-nivolumab
	NCT04699188	I/II	refractory	JDQ443+/-TNO155+/-spartalizumab
	NCT03668431	II	refractory	Dabrafenib-trametinib-spartalizumab
Bispecific antibodies	NCT04826003	I	refractory	RO7122290-cibisatamab-obinutuzimab
	NCT04468607	I	refractory	BLYG8824A

### 3.1 Immune checkpoint inhibitor combinations

Immune checkpoint inhibitors lead to impressive, deep, and long-lasting responses in MSI mCRC in both the first-line and the refractory setting (12, 74). However, the results in the MSS population have not replicated those observed in the MSI setting. The anti-PD1-antibody pembrolizumab was evaluated in a phase II clinical trial which included 11 patients with MSI tumors and 21 MSS refractory tumors. The overall response rate (ORR) was 0% (95% confidence interval [CI], 0-20), and the PFS rate at 20 weeks was 11% (2 of 18 patients; 95% CI, 1-35). Somatic mutations per tumor, neoantigen load and CD8 infiltration were significantly lower in patients with MSS tumors suggesting that a hot-tumor microenvironment -not observed in MSS tumors- is needed to achieve immune response. Similar results were obtained in the phase I trial evaluating nivolumab in solid tumors, which included 19 patients with MSS tumors in the expansion cohort (75). Only one patient with CRC had a PD-L1 positive tumor and this patient did not respond. The combination of nivolumab and the anti-CTLA4 ipilimumab was also tested in the phase II CheckMate-142 trial. Overall, 23 patients with MSS tumors were enrolled; median PFS was 1.4 months, highlighting the lack of clinical activity of the double immune checkpoint combination (46). These results suggest there is no meaningful clinical activity with combined PD-1 and CTLA4 blockade in MSS mCRC, although a small number of patients achieved some clinical benefit (47).

Dual blockade of PD-1 and LAG-3 (an immune checkpoint inhibitor mainly expressed on T cells) has the potential to synergistically restore T-cell functionality, and therefore to boost the immune system antitumor activity (76, 77). The phase I trial (NCT03156114) evaluated the combination of BI754111 (an anti-LAG-3 monoclonal antibody) and BI754091 (an antiPD-1) in patients with advanced solid tumors. In the mCRC cohort, 40 patients with refractory tumors were included with an ORR of 7.5%, and a DCR of 35% (49). Other ongoing strategies include the combination of the LAG-3 inhibitor relatlimab with an anti-PD1 (NCT0306422067 and NCT05064059) (48) ⁠with the same results compared with immunotherapy alone.

More recently, promising activity has been seen in MSS tumors with novel immune checkpoint inhibitor combinations. Botensilimab, a novel innate/adaptive immune activator (Fc-enhanced CTLA4-inhibitor) in combination with balstilimab (anti-PD1) were tested in the first-in-human, phase I C-800 trial. The CRC MSS cohort included 41 heavily pretreated patients, and an impressive ORR of 24% and a disease control rate (DCR) of 73% was seen, with a well-tolerated safety profile (no grade 4 or 5 adverse events were reported). The exploratory analysis in terms of liver involvement showed enriched responses among the 24 patients without active liver metastases (ORR 42%, DCR 96%) suggesting that liver metastases may preclude immune system activation (8, 78, 79). Although the classical approach with immune-checkpoint inhibitors blockade has not been demonstrated to be as effective as in the MSI scenario, novel combinations nonetheless demonstrate promising activity, particularly among well-selected patients without liver metastases.

### 3.2 Immune checkpoint inhibitors in combination with MEK inhibitors

Activation of the MAPK pathway leads to proliferative tumor effects as well as decreased T cell infiltration and immunosuppression (38, 39). In preclinical models, MEK inhibition resulted in induced INFγ-dependent HLA and PDL1 upregulation, suggesting the potential synergic effect of MEK inhibitors with immunotherapy (80, 81). MEK inhibition also profoundly blocked naive CD8 T cell priming in tumor-bearing mice; however, it increased the number of effector-phenotype antigen-specific CD8 T cells within the tumor. In addition, MEK inhibition decrease tumor-infiltrating CD8 T cells from death driven by prolonged T cell receptor stimulation while sparing cytotoxic activity (81).

The phase II randomized CO.26 trial evaluated the anti-PD1 durvalumab plus the MEK inhibitor tremelimumab compared with best supportive care. There were no significant differences in median PFS (1.9 and 1.8 months respectively). However, longer overall survival (OS) was observed among patients treated with durvalumab plus tremelimumab (HR, 0.66; 90% CI, 0.49-0.89; P = .02) with a particular benefit among patients with plasmatic TMB >28 variants per megabase. Atezolizumab plus the MEK inhibitor cobimetinib combination was evaluated in a phase Ib trail which included 84 patients with refractory CRC resulting in an ORR of 8% (82). Besides, a phase 3 trial randomized patients with refractory CRC to receive either atezolizumab plus cobimetinib, atezolizumab in monotherapy, or regorafenib. The trial did not meet its primary endpoint. Median OS was 8.9 months for the experimental combination therapy arm, 8.5 months for the control arm with regorafenib, and 7.1 months for patients receiving atezolizumab (50). These negative results can be partially explained by the enrollment of patients with refractory mCRC, without selecting by *RAS/BRAF* mutational status, and the fact that single MEK blockade fails to maintain MAPK inhibition due to adaptive feedback throughout EGFR receptor (83, 84).

### 3.3 Combination of immune checkpoint inhibitors with tyrosine kinase inhibitors

Various combinations with immune checkpoint inhibitors and tyrosine kinase inhibitors have been investigated suggesting that tyrosine kinase inhibitors, particularly angiogenesis inhibitors, may decrease tumor-associated macrophages and Tregs, and enhance T cell infiltration and activation, as well as increase dendritic cell maturation increasing tumor antigenicity and tumor immunogenicity (85, 86).

The REGONIVO phase Ib study in Asia evaluated regorafenib in combination with nivolumab in patients with MSS and MSI-H CRC who had progress on previous systemic chemotherapy (34). Patients were treated with regorafenib and nivolumab. The ORR among patients with MSS disease was 33%, median PFS was 7.9 months, and median OS was not reached. The one-year PFS rate was 42%, suggesting durable disease control. However, among patients with liver metastasis, the ORR was 8%, whereas it was 64% for patients with lung metastasis, again suggesting that liver metastases may play an immunosuppressive role hampering immunotherapy activity. Other studies (NCT03712943 ⁠and NCT04126733) have investigated the same combination with median PFS of 4.3 and 1.8 months respectively (51, 52). Regorafenib was also combined with pembrolizumab in a phase I/II study in patients with refractory MSS CRC. No objective responses were observed. Median PFS was 2 months and median OS was 10.9 months (53). Another combination of regorafenib with another anti-PD-L1 antibody avelumab in a phase II trial examined the efficacy and safety of this combination in 48 patients with mismatch-repair-proficient CRC. The trial did not show any objective responses, however 23 patients presented stable disease (54%), and median PFS was 3.6 months (95% CI 1.8–5.4), and median OS was 10.8 months (95% CI 5.9–non-achieved-NA). Correlative analysis suggested an association between high tumor-associated macrophages and poor outcomes, whereas increased CD8 T cell infiltration suggested an improvement in clinical outcomes (54). Furthermore, a phase I trial combining regorafenib with ipilimumab and nivolumab in patients with MSS CRC (NCT04362839) gave an ORR of 31%, with a median PFS of 4 months. Cabozantinib was combined with durvalumab in a prospective, open-label, multicenter phase II trial giving a 28% ORR, median PFS of 4.4. months, and OS of 9.1 months (55). Of note, cabozantinib was also evaluated in combination with atezolizumab in the COSMIC-021 phase 1b trial which included 31 patients with MSS, refractory mCRC. Patients with wild-type *RAS* had numerically longer PFS and OS and higher ORR compared with patients with *RAS* mutated tumors (5.8 vs 2.7 months, 16.7 vs 8.7 months and 25% vs 0% respectively) (56). The pembrolizumab and lenvatinib combination in the non-randomized, phase II, LEAP-017 trial showed promising antitumor activity with an ORR of 22% and median PFS of 2.3 months among patients with refractory MSS CRC (58).⁠⁠ Finally, the combination of ibrutinib, a Bruton’s tyrosine kinase (BTK) inhibitor, has also been tested in mCRC in combination with pembrolizumab showing poor clinical activity (ORR 0%, mPFS 1.4, and mOS 6.6 months) (57).

CRC harboring the *BRAF*-V600E mutation deserves specific attention. With its well-known poor prognostic, the BEACON trial was the first phase III trial demonstrating an advantage with the BRAF inhibitor encorafenib plus cetuximab with or without the MEK inhibitor binimetinib. Correlative analysis from paired biopsies demonstrated an increase in T cell infiltration and cytotoxic infiltration after the initiation of a BRAF inhibitor, suggesting potential cooperation between BRAF-targeting and the immune response (43). This is supported given that the combination of immune checkpoint inhibitors plus BRAF inhibitors has shown promising results: the phase I trial combining dabrafenib-trametinib and spartalizumab showed a 33% ORR with a 76% of DCR. In the same setting, the SWOG phase I/II trial demonstrated promising activity of the encorafenib-cetuximab-nivolumab triple combination, with an ORR of 50%, DCR of 95% and impressive 7.4 month median PFS and 15.1 month median OS in *BRAF*-V600E mutated MSS mCRC (42).

### 3.4 Immunotherapy in combination with anti-EGFR agents

Targeting EGFR signaling—particularly the MAPK pathway—holds promise for creating synergy with immune checkpoint inhibitors, alone or in combination with chemotherapy. Anti-EGFR activity leads to NK cell activation and subsequent lytic activity on tumor cells by antibody-dependent cellular cytotoxicity, Treg immunosuppression and induces PD-L1 expression on tumor cells *via* INFγ (87–89). Panitumumab in combination with nivolumab and ipilimumab, has been investigated in a phase II study in patients with *RAS/RAF* wild-type mCRC.⁠ The ORR was 5%, with a median PFS of 5.7 months, favoring this triplet regimen over single-agent panitumumab (59)⁠. The combination of avelumab with cetuximab has been explored in the third-line chemorefractory setting in *RAS* wild-type mCRC with no selection regarding microsatellite status, in patients who had a complete or partial response to first-line chemotherapy plus anti-EGFR drugs. A promising median OS of 11.6 months and median PFS of 3.6 months were achieved (60).⁠ Together these data suggest potential synergism between immune checkpoint inhibitors and anti-EGFR drugs.

### 3.5 Immunotherapy in combination with chemotherapy

⁠Combined chemotherapy and immunotherapy have been tested in several clinical trials. The rationale for these combinations relies on the fact that chemotherapy promotes immune cell infiltration, and dendritic cell maturation, enhances antigen presentation, as well as inhibits immunosuppressive cells (90–92). The addition of avelumab to FOLFOX and cetuximab in this setting was explored in the single-arm, phase II AVETUX trial which included 42 patients (2 MSI, 40 MSS). With RAS/BRAF wild-type tumors. Although the combined treatment gave an ORR of 79.5%, the PFS rate at 12 months was 40%, and the study did not reach its primary endpoint (12 months PFS 57%) (61). AtezoTRIBE, a randomized phase II trial, investigated the combination of FOLFOXIRI and bevacizumab, with or without atezolizumab, in patients with mCRC regardless of MSS/MSI status. Among the MSS subgroup, PFS improved from 11.4 to 12.9 months in patients treated with chemotherapy combined with atezolizumab (62)⁠. The BACCI phase II trial evaluated the addition of atezolizumab or placebo to capecitabine and bevacizumab in the chemo-refractory setting regardless of MSS status. The trial achieved its primary endpoint of median PFS (4.4 vs 3.6 months) in the overall population with a non-significant PFS improvement among the MSS subgroup (5.3 vs 3.3 months) (93).⁠⁠ Another study, the phase III CheckMate9X8, evaluated the addition of nivolumab to standard first-line chemotherapy with FOLFOX-bevacizumab vs FOLFOX-bevacizumab in patients with previously untreated, unresectable, mCRC. However, the primary endpoint of PFS was not met, with the same median PFS of 11.9 months in both arms (64)⁠.⁠ Finally, the phase II MEDITREME trial evaluated the efficacy and safety of mFOLFOX6 (6 cycles) in combination with durvalumab and tremelimumab as induction therapy followed by maintenance therapy with durvalumab in patients with previously untreated *RAS*-mutated mCRC. ORR was 61% and mPFS was 8.4 months. Biomarker analysis showed that high baseline levels of Th2 and PDL1+ MDSC were associated with poor PFS (66).

After first-line induction therapy with FOLFOX-bevacizumab maintenance treatment with 5-FU plus atezolizumab has been also evaluated in the phase II MODUL trial which included patients with metastatic *BRAF* wild-type CRC. However, the trial did not show a significant difference in either PFS or OS (67). Results are awaited for the ongoing phase Ib/II COLUMBIA-1 trial is comparing FOLFOX and bevacizumab in combination with durvalumab and the anti CD37 oleclumab versus FOLFOX and bevacizumab (NCT04068610).

Also combining chemotherapy plus immunotherapy, the phase II MAYA trial (NCT03832621) evaluated the effect of temozolomide on TMB in terms of response to immune checkpoint inhibitors among MSS mCRC patients. Patients with chemo-refractory CRC were prescreened for MSS status and MGMT silencing. Eligible patients received temozolomide followed, in the absence of progression, by combination with ipilimumab and nivolumab. After a median follow-up of 23.1 months, the 8-month PFS rate was 36% and median PFS and OS were 7.0 and 18.4 months, respectively, with an ORR of 45% (65). Likewise, the ⁠ARETHUSA (NCT03519412) phase II trial is enrolling patients with MSS, *RAS* mutant CRC tumors that are MGMT negative and promoter methylation-positive and have TMB >20 mut/Mb after treatment with temozolomide. After this priming phase with temozolomide, patients with high TMB are treated with pembrolizumab (94) based on evidence that temozolomide can increase mutational burden in both tumor tissue and blood of CRC patients.

### 3.6 ⁠Bispecific antibodies

Bispecific antibodies are an large engineered family of molecules designed to recognize two different epitopes or antigens simultaneously, aiming to enhance the host’s immune activity against tumor cells by binding both tumor-enriched antigens (e.g., CEA, CEACAM, or CD276 antigen) and immune cells (mainly T cells using the CD3 receptor) (95).⁠ Cibisatamab (RO6958688, also known as CEA-TCB) is a T cell bispecific antibody targeting both CEA on tumor cells and CD3 on T cells, leading to crosslinking of tumor cells and lymphocyte T cell engagement and activation. In preclinical models, cibisatamab⁠ demonstrated deep antitumor activity, leading to increased intratumoral T cell infiltration and activation and PD-1/PD-L1 upregulation. In two ongoing dose escalation phase I studies, cibisatamab monotherapy or combined with atezolizumab, is administrated in patients with advanced CEA-positive solid tumors. The monotherapy group included 80 patients (70 patients with CRC), and the combination group included 45 patients (35 with CRC). CT scans revealed signs of tumor inflammation flare within 48 hours of the first dose. Additionally, tumor reduction was seen in MSS tumors in 13% and 36% of CRC patients in the cibisatamab monotherapy and combination groups respectively with an impressive DCR of 45% and 82%, respectively. Among patients with mCRC 68 patients treated with cibisatamab and 38 patients treated with cibisatamab in combination with atezolizumab, 10 (28%) and 6 (60%) respectively presented a metabolic partial response by PET scan 4-6 weeks after treatment initiation with evidence of antitumor activity during dose escalation (68).

### 3.7 ⁠Immunotherapy combined with radiotherapy, and novel vaccines and intratumoral therapies

Radiation therapy is an effective treatment for many cancers and a part of the multidisciplinary approach to cancer care, exploiting the mechanism of damage of DNA inside cancer cells. Distantly, radiotherapy can induce an abscopal effect (i.e., tumor shrinkage), which might results in tumor shrinkage outside the irradiated field due to an increase of immune cell tumor infiltrates and direct presentation of tumor antigens (96, 97).⁠⁠⁠ Durvalumab plus tremelimumab combined with radiotherapy in patients with mCRC was evaluated in a single arm phase II trial. ORR was 8.3%, and median PFS and median OS were 1.8 and 11.4 months respectively (69). The radiotherapy with ipilimumab and nivolumab combination has also been tested, leading to shrinkage of non-irradiated distant tumors with an ORR of 12.5% (70).

In a more recent approach, cancer vaccines may play a role in enhancing the immune system. stimulating tumor antigen-specific cytotoxic T lymphocytes that recognize and eliminate cancer cells (98).⁠ This strategy was evaluated in a randomized phase II trial with two groups of patients, one treated with the best supportive care and the other with autologous dendritic cells plus best supportive care (71)⁠. The study demonstrates that this antibody-drug conjugate generates a tumor-specific immune response but did not provide a survival benefit (median PFS was 2.7 vs 2.3 respectively and median OS was 6.2 vs 4.7, respectively). Another phase II trial evaluated the combination of a five peptides vaccine derived from tumor-associated antigens with oxaliplatin-based chemotherapy in the first-line setting, demonstrating the generation of a tumor-specific immune response but again without meaningful clinical benefit (72).⁠

Finally, among novel cancer treatment strategies, oncolytic virotherapy has shown encouraging progress. Genetically modified herpes simplex viruses (HSVs) have demonstrated to induce cytolytic cell death and liberation of progeny virions, which infect adjacent tumor cells without adversely affecting untransformed parent cells. A phase I/II clinical trial genetically engineered oncolytic HSV NV1020 (73)⁠. Among the 22 patients treated, 50% showed stable disease after NV1020 administration with a 68% DCR and median OS of 11.8 months after treatment with chemotherapy.

## 4 Discussion

Unlike MSI mCRC, the efficacy of immune checkpoint inhibitors alone or in combination with other therapeutics is limited to a narrow subgroup of patients. While the vast majority of MSS CRC tumors will not respond to immunotherapy, there appears to be a borderline immunogenic tumor subgroup that manifest with stable disease and partial responses. Classic predictive biomarkers such as PDL-1 expression or TMB do not appear to be valuable biomarkers for efficacy in MSS CRC. The main reasons for the lack of response to immunotherapy in MSS mCRC are the tumor cell infiltrate which has low levels of TIL and high infiltration of T cell suppression, Tregs, and macrophages. Other factors influencing poor outcomes are low TMB and low immunogenicity of MSS tumor-related neoantigens and genomic and clinical characteristics including a high rate of WNT/β-catenin pathway mutations, and the presence of liver metastases. These features highlight the non-inflamed reality of MSS tumors reflecting a low immunogenic environment. However, the tumor microenvironment is amenable to be modulated with other therapeutics that may increase immune checkpoint inhibitor responses. Furthermore, the genomic landscape of MSS CRC, mostly driven by the WNT/β-catenin pathway, has been identified as a critical factor of immunosuppression and T cell antitumor activity impairment, limiting immune checkpoint inhibitor activity. As a result, there are currently several clinical trials aiming to inhibit WNT/β-catenin pathway signaling using combination therapies with immune checkpoint inhibitors. Taking a similar approach, some studies have demonstrated that liver metastases promote an immunosuppressive microenvironment that limits immune checkpoint efficacy. Considering these upfront barriers that hamper immunotherapy activity, several combinations have been tested in combination with immune checkpoint inhibitors, including tyrosine kinase inhibitors, anti-EGFR, chemotherapy, and radiotherapy, while novel approached including bispecific antibodies, ADCs, and vaccines, with modest improvements in terms of clinical outcomes. Currently there are several ongoing trials assessing new drug combinations that aim to overcome the acquired mechanism of resistance in MSS CRC, as summarized in **Table 2**. These data form the basis of a better understanding of the biology underlying MSS colorectal tumors that will lead to improved clinical outcomes and the identification of clinical biomarkers relevant to this population. Recently several trials demonstrated that outcomes in patients with MSS CRC can be improved with either better designed drugs, such as botensilimab and balstilimab, or with bispecific antibodies, or with a smart trial design such as the MAYA or the ARETHUSA trials in which the TMB is successfully modulated using chemotherapy to boost the effect of subsequent immune checkpoint inhibitors. Similarly, liver metastases, mostly enriched in TGF-β, have been identified as a negative biomarker of response using immunotherapy combinations in several clinical trials. Besides, other treatment strategies are being developed for MSS CRC such as neoadjuvant immunotherapy. Indeed, the NICHE trial demonstrated that immune checkpoint inhibitors may have a role during early-stage disease in MSS CRC. The NICHE trial showed 27% pathological responses among patients with early-stage, low TMB, MSS, CRC treated with neoadjuvant nivolumab and ipilimumab combination. Of note, correlative analysis showed that CD8+ PD1+ cell infiltration was predictive of response (99). Despite these promising results, larger studies and longer follow-up are still required. Ultimately, a deep understanding of mechanisms of immunotherapy resistance and the heterogenous spectrum of mCRC is needed in order to improve therapeutical co-strategies to overcome primary resistance to immunotherapy. Furthermore, biomarker identification may help for an optimal patient selection.

## Author contributions

JR, and FB wrote the first draft of the manuscript. All authors contributed to the manuscript revision. EE supervised the final version. All authors contributed to the article and approved the submitted version.
